# The role of prefrontal cortex in a moral judgment task using functional near‐infrared spectroscopy

**DOI:** 10.1002/brb3.1116

**Published:** 2018-09-25

**Authors:** Hadis Dashtestani, Rachel Zaragoza, Riley Kermanian, Kristine M. Knutson, Milton Halem, Aisling Casey, Nader Shahni Karamzadeh, Afrouz A. Anderson, Albert Claude Boccara, Amir Gandjbakhche

**Affiliations:** ^1^ Section on Analytical and Functional Biophotonics National Institute of Child Health and Human Development, National Institutes of Health Bethesda Maryland; ^2^ Department of Computer Science and Electrical Engineering University of Maryland Baltimore County Baltimore Maryland; ^3^ Brain Neurology Unit National Institute of Neurological Disorders and Stroke National Institutes of Health Bethesda Maryland; ^4^ Institut Langevin, ESPCI Paris PSL Research University Paris France

**Keywords:** dorsolateral prefrontal cortex, functional near‐infrared spectroscopy, mixed effect model, moral judgment

## Abstract

**Background:**

Understanding the neural basis of moral judgment (MJ) and human decision‐making has been the subject of numerous studies because of their impact on daily life activities and social norms. Here, we aimed to investigate the neural process of MJ using functional near‐infrared spectroscopy (fNIRS), a noninvasive, portable, and affordable neuroimaging modality.

**Methods:**

We examined prefrontal cortex (PFC) activation in 33 healthy participants engaging in MJ exercises. We hypothesized that participants presented with personal (emotionally salient) and impersonal (less emotional) dilemmas would exhibit different brain activation observable through fNIRS. We also investigated the effects of utilitarian and nonutilitarian responses to MJ scenarios on PFC activation. Utilitarian responses are those that favor the greatest good while nonutilitarian responses favor moral actions. Mixed effect models were applied to model the cerebral hemodynamic changes that occurred during MJ dilemmas.

**Results and conclusions:**

Our analysis found significant differences in PFC activation during personal versus impersonal dilemmas. Specifically, the left dorsolateral PFC was highly activated during impersonal MJ when a nonutilitarian decision was made. This is consistent with the majority of relevant fMRI studies, and demonstrates the feasibility of using fNIRS, with its portable and motion tolerant capacities, to investigate the neural basis of MJ dilemmas.

## 1 INTRODUCTION

Moral judgment (MJ) is the process of evaluating what is right or wrong based on social norms (Jonathan, 2003; Prehn et al., 2007). Many studies have presented subjects with MJ scenarios and follow‐up questions to prompt decision‐making, in which a subject chooses a theoretical course of action; some of these studies have shown that moral judgments are not based solely on rational thoughts but also on emotions (Glenn, Raine, & Schug, 2009; Greene et al., 2004, 2001; Han, Chen, Jeong, & Glover, 2016; Han, Glover, & Jeong, 2014; Koenigs et al., 2007; Prehn et al., 2007). Greene et al. (2004) classified MJ scenarios as either personal or impersonal MJ. If subjects relied on more emotional processing to make a decision, those scenarios were considered to be personal MJ scenarios (Greene et al., 2004); if subjects relied on more cognitive processing, those scenarios were considered to be impersonal MJ. Here, we used the classification system.

The classic Trolley Dilemma describes an impersonal MJ scenario in which a trolley is hurtling toward five workers on the track. One option presented is to flip a switch to divert the course of the trolley, which would result in the trolley hurtling toward one person on the opposite side of the track, killing this one person. The other is to do nothing and allow the five workers to die. In this scenario, studies show that most people respond that it is morally acceptable to flip the switch and save five lives at the expense of one. This is called utilitarian decision‐making, where a theoretical course of action is chosen to benefit the most number of people regardless of how immoral the action itself may be (Foot, 1978; Thomson, 1986). An alternative personal MJ scenario, called the Footbridge Dilemma, describes a trolley hurtling toward five people on the track. The participant can either push a man off a footbridge, in which his body weight would stop the course of the trolley and save five lives, or do nothing and allow five people to die. In this scenario, most people choose not push the one man off the footbridge (Thomson, 1986), refusing to be directly responsible for one death at the expense of five indirectly. This is a nonutilitarian decision, as a moral action with a less beneficial outcome is chosen over an immoral action with a better outcome (Greene et al., 2001).

Functional imaging studies on nonpatient (control) populations involving MJ (Han, 2017, 2015; Han et al., 2016; Heekeren, Wartenburger, Schmidt, Schwintowski, & Villringer, 2003; Moll & Oliveira‐Souza, 2007) and moral reasoning (Borg et al., 2006; Greene et al., 2004, 2001 ) have detected consistent activations of the orbitofrontal and ventromedial prefrontal cortex (VM‐PFC). According to the dual‐process theory of MJ, Greene posited that emotional and cognitive processes are competing systems during MJ decision‐making (Greene et al., 2004; Greene, 2007; Han, 2017; Han et al., 2016, 2015 ). He also hypothesized that the VM‐PFC is responsible for emotional engagement during moral judgment of personal scenarios resulting in nonutilitarian decision‐making, while the dorsolateral PFC (DL‐PFC) is responsible for utilitarian (logical) judgments (Glenn et al., 2010; Glenn, Raine, Schug, Young, & Hauser, 2009; Greene et al., 2004; Hutcherson, Montaser‐Kouhsari, Woodward, & Rangel, 2015) that are thought to engage more cognitive processes and fewer emotional processes. This further supports the idea that the VM‐PFC may be involved in processing emotionally salient events, whereas the DL‐PFC is thought to be responsible for more goal‐direct behaviors. Meta‐analyses have also shown similar areas of activation during moral tasks. Eres, Louis, and Molenberghs (2018); Han (2017), conducted meta‐analyses on fMRI datasets using activation likelihood estimation (ALE) and found the medial prefrontal cortex and lateral orbitofrontal cortex are the common brain regions highly activated during MJ dilemmas.

All of the above studies and many of the others that have attempted to determine the neural basis for moral decision‐making have used fMRI; however, functional near‐infrared spectroscopy (fNIRS) is a modality well suited for such a task. fNIRS is a highly promising neuroimaging modality that provides an efficient way to continuously monitor changes in blood oxygenation in the cerebral cortex (Franceschini, Fantini, Thompson, Culver, & Boas, 2003) In addition, its portability and high tolerance to patient movement make it optimal for use in nonclinical environments, such as jails, or on special subject populations ill‐suited for fMRI scan requirements, such as children. One drawback of this modality is that it can detect hemodynamic activity only from the brain cortex, which is also common in some other neuroimaging modalities such as electroencephalography. Nonetheless, its many practical aspects make it an attractive diagnostic tool for neurological disorders characterized by altered brain activation. For instance, Strait & Scheutz (Strait & Scheutz, 2014) used fNIRS and MJ scenarios to investigate the effects of agency and personal incentive in the PFC.

In the present study, we hypothesized that differential brain activation would be observed through fNIRS during judgment of personal versus impersonal dilemmas. Specifically, we included nonpatient adult participants who were presented with personal and impersonal dilemmas. We anticipated that these different types of scenarios would elicit differential brain activation observable through fNIRS. We also investigated the effects of utilitarian compared to nonutilitarian responses on prefrontal brain activation.

Overall, this study in normal controls is our first step in determining the efficacy of fNIRS in detecting PFC activity during the MJ task, while our plan is to eventually use fNIRS on a psychiatric population. Studies using fMRI have found PFC dysfunction in conjunction with distinct patterns of brain activation in some psychiatric disorders including antisocial personality disorder and conduct disorder (Contreras‐Rodríguez et al., 2015; Fede et al., 2016; Geurts, 2016; Glenn et al., 2009; Yang et al., 2015; Yoder, Harenski, Kiehl, & Decety, 2015). Additionally, it has been shown that moral judgment (MJ) is impaired in individuals suffering from these disorders (Blair, 1995; Fede et al., 2016; Gao & Tang, 2013; Geurts et al., 2016; Glenn et al., 2009; Koenigs, Kruepke, Zeier, & Newman, 2011; Seara‐Cardoso, Dolberg, Neumann, Roiser, & Viding, 2013; Yoder et al., 2015; Young, Koenigs, Kruepke, & Newman, 2012). Our plan is to eventually apply fNIRS on this psychiatric population in order to determine if they have differentiable functional activity during MJ tasks when compared to normal controls.

## 2 MATERIALS AND METHODS

### 2.1 NIRS data acquisition

**Figure 1:**
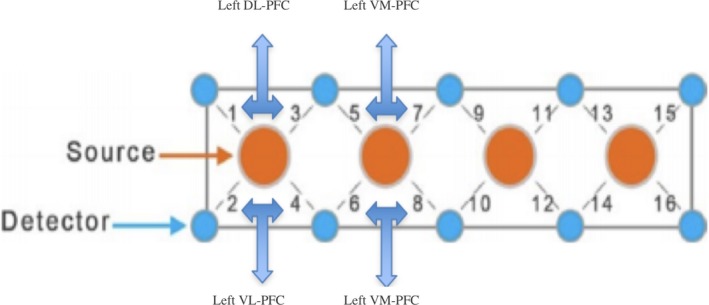
The configuration of probes for the fNIRS device. There are four sources and 10 detectors resulting in 16 source/detector (channels) pairs

fNIRS is an imaging modality that uses near‐infrared light (700–1,000 nm) to measure changes in blood oxygenation. We used an fNIRS Model 1,000 (fNIRS Devices LLC, Potomac, MD, USA). The lights were emitted from each source at 730 and 850 nm wavelengths. The system had four sources and ten detectors, with a source‐detector separation of 2.5 cm, for a total of 16 channels of oxyhemoglobin (HbO) and deoxyhemoglobin (HbR). The sampling frequency was 2 Hz. The channel arrangement can be seen in Figure 1. The headband was always placed by one of two trained experimenters, who aligned the center between optodes 8 and 10, with nasion.

### 2.2 Experiment design

**Figure 2:**
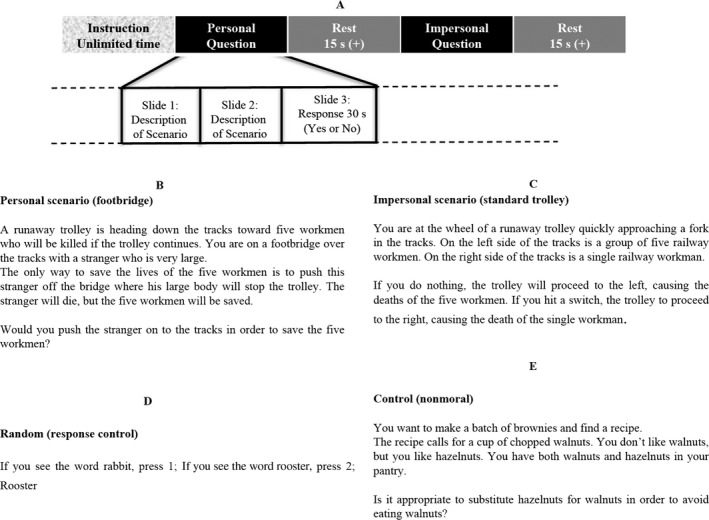
(a) The MJ paradigm for this study. Each question consisted of three slides: the first two slides described a scenario, the third one included a MJ question in which subject had 30 s to respond, and then a 15 s resting period. The participant answered “Yes” or “No” by pressing “1” or “2” on the keyboard, respectively. “Yes” indicated they were for the action presented. (b) Shows a sample personal scenario, which has a utilitarian response. (c) Shows an impersonal scenario, which also has a utilitarian response. (d) To control for random responses, subjects were asked to press “1” if they saw one word and press “2” if they saw another word. (e) Nonmoral control questions. (d) and € ensured the subject was paying attention and reading the scenarios throughout the task. Accuracy on these slides controlled for random responses and fatigue. (f) Shows an example of the three slides presented to participants in this MJ task

This experiment was modeled after the study conducted by Greene et al. (2004). We adopted 21 personal and 14 impersonal MJ exercises from their studies. Furthermore, we added five nonmoral control exercises and five random questions to control for responses and fatigue. Each exercise consisted of three slides: the first two slides described a scenario, and the third one included a MJ question in which subject had 30 s to respond, followed by a 15 s resting period. The participant answered “Yes” or “No” by pressing “1” or “2” on the keyboard, respectively. “Yes” indicated they were for the action presented. Figure 2a shows a timing diagram of the task, Figure 2b–e illustrate a sample of personal, impersonal, random, and control scenarios, respectively. All the moral judgment questions can be found in the Supporting Information Appendix S1. Moreover, Supporting Information Figures S1 and S2 in Appendix S1 show the order of scenarios and a sample of three slides. The order of the questions was pseudorandom. The task was developed using E‐Prime 2.0 software (Psychology Software Tools, Pittsburgh, PA, USA).

### 2.3 Participants

A total of 33 healthy subjects (15 males) age 18–58 (mean 33.7) with no history of concussions or psychological and neurological disorders participated in the task. Every participant had normal or corrected vision. Their handedness was assessed by the Edinburgh Inventory (Oldfield, 1971) questionnaire. Thirty‐one participants were right handed, two were ambidextrous, and one was left handed. All participants gave written informed consent prior to the experiment, which was performed in compliance with the Declaration of Helsinki and approved by the National Institute of Child Health and Human Development’s Institutional Review Board.

### 2.4 Preprocessing and artifact removal

The hemodynamic changes for each of the 16 channels were calculated using the Modified Beer Lambert Law (MBLL) (Hiraoka et al., 1993). As stated in (Bauernfeind, Wriessnegger, Daly, & Müller‐Putz, 2014), the frequency of pulse waves is typically around 1–2 Hz, Mayer waves frequency is around 0.1 Hz, and the respiration frequency is around 0.3 Hz (Anderson et al., 2017; Greve et al., 2009; Sherafati, Eggebrecht, Bergonzi, Burns‐Yocum, & Culver, 2018). Here, HbO signals were low passed filtered at 0.1 Hz, then the moving average filter with 1.5 s timing window was applied to smooth the signal. Subsequently, the linear and nonlinear trends were removed by fitting a low order (order of 6) polynomial to the fNIRS signals and subtracting it from the original signal (Karamzadeh et al., 2016; Minati, Visani, Dowell, Medford, & Critchley, 2011; Pfeifer, Scholkmann, & Labruyère, 2017; Zhao, Ji, Li, & Li, 2018).

Next, we extracted fNIRS segments using their corresponding markers. We only considered changes in the HbO in our analysis. It has been shown in studies comparing fMRI and fNIRS that changes in HbO signal are better correlated with BOLD fMRI signal and brain activation than HbR (Greve et al., 2009; Sato et al., 2013; Strangman, Culver, Thompson, & Boas, 2002), and that HbO signal has higher sensitivity to changes in cerebral blood flow (Hoshi, 2003; Lindenberger, Li, Gruber, & Müller, 2009; Zhang, Liu, Pelowski, Jia, & Yu, 2017).

Following (McKendrick, Ayaz, Olmstead, & Parasuraman, 2014), we assigned two or four channels to specific prefrontal regions. These regions approximately represented left DL‐PFC, left ventrolateral PFC (VL‐PFC), left VM‐PFC, right VM‐PFC, right DL‐PFC, and right VL‐PFC (Figure 1).

### 2.5 Data analysis: statistical model

Mixed effect models were used to assess changes in HbO as a function of category (personal or impersonal scenario), brain regions and responses. The traditional way to run a repeated measure analysis is to consider each trial as a multivariate task and each response as a separate variable. For our experiment, we preferred a mixed effect model over repeated measures ANOVA. Mixed effect models do not require the same number of observations per subject; therefore, residual maximum likelihood (REML) can be applied to unbalanced designs (such as our 21 personal and 14 impersonal dilemmas). Using mixed effect models, we were able to find the unique intercept and slope of estimation for each subject. In other words, we estimated the parameters unique to individual participants. Moreover, while the default approach to deal with missing data in conventional statistical models is to drop observations with missing values, the mixed effect models use regression techniques to estimate missing data (Krueger & Tian, 2004; Stiratelli, Laird, & Ware, 1984). Analyses were performed in R using REML in package lme4 in R (Bates, Maechler, & Bolker, 2007).

For our first hypothesis, we investigated whether the hemodynamic response to personal dilemmas could be distinguished from the hemodynamic response to impersonal dilemmas through fNIRS. Our fitted model took average HbO changes as a dependent variable, and used the category of dilemma, either personal or impersonal, as an independent variable and subject as a random effect. Denominator degrees of freedom for the *t* test were calculated based on Satterthwaite approximation (Schaalje, McBride, & Fellingham, 2002). To identify the sources of significant differences (*p* < 0.05) in the pairwise comparisons, we used the multcomp package in R, which performs multiple comparisons under the parametric model framework. Specifically, the glht function, whose core functionality is to apply single‐step comparison tests, was used. The glht function takes a fitted estimated model and a hypothesis matrix to perform multiple comparisons.

We used the Tukey method, one of the best methods for controlling Type I error rate in pairwise post hoc tests (Tukey, 1949). The single‐step method which is more powerful than Bonferroni correction method (Bretz, Hothorn, & Westfall, 2016) was applied to control for multiple comparisons and adjustments (family‐wise error, *p* < 0.05). Table 1 shows more details of the different models we implemented.

**Table 1:** Significant effects of different factors on average HbO changes

Row	Fixed effects	CPCT	df_num_	df_den_	*F* value	$\Omega_{0}^{2}$	Pr (>F)
Hemodynamic changes as a function of category (personal vs. impersonal MJ)							
A	Fixed effect: category		1.00	6,854.1	4.4795	0.402	0.03434*
Hemodynamic changes as a function of category (personal vs. impersonal MJ) in PFC regions							
B	Fixed effect: category × region	2	5.00	6,850	3.1743	0.434	0.007266**
Hemodynamic changes as a function of category (personal vs. impersonal MJ) and response (utilitarian vs. nonutilitarian) in PFC regions							
C	Fixed effect: category × region × response	3	23.00	6,836.4	1.5545	0.446	0.04409*
Hemodynamic changes as a function of response (utilitarian/nonutilitarian) in personal MJ							
D	Fixed effect: response		1.00	3,977.3	−1.986	0.463	0.0471*
Hemodynamic changes in PFC regions, in impersonal MJ							
E	Fixed effect: region	4	5.00	2,719	4.1423	0.809	0.000945***
Hemodynamic changes in PFC regions considering responses (utilitarian/nonutilitarian), in impersonal MJ							
F	Fixed effect: region × response	5	11.00	6,847.4	1.8639	0.826	0.0391*

Notes

CPCT: Corresponding Pairwise Comparison Table.

Test of fixed effects: Denominator degrees of freedom (df_den_) were calculated with Satterthwaite approximation. Only results with *p* < 0.05 are reported.

*p < 0.05.

**p < 0.01.

***p < 0.001.

Another model was built to determine activation patterns in different prefrontal areas as a function of the MJ exercises. In this model, prefrontal brain regions and the personal/impersonal scenarios were considered independent variables, while dependent variables and random effects remained the same as in the first model. Finally, we tested how utilitarian and nonutilitarian decisions regarding MJs would affect different prefrontal regions’ hemodynamic responses.

Then, we focused our research to separate analyses of personal and impersonal MJ. All the above models were rebuilt using either personal or impersonal moral dilemmas.

In order to calculate the effect size in mixed effect models as Bates, Mächler, Bolker, and Walker (2014) pointed out, there is not an agreed upon method for the inclusion or exclusion of the random effects variances. As suggested by Xu (2003), we calculated $\Omega_{0}^{2}$ defined as model total variation. Table 1 shows the result for each model.

## 3 RESULTS

**Figure 3:**
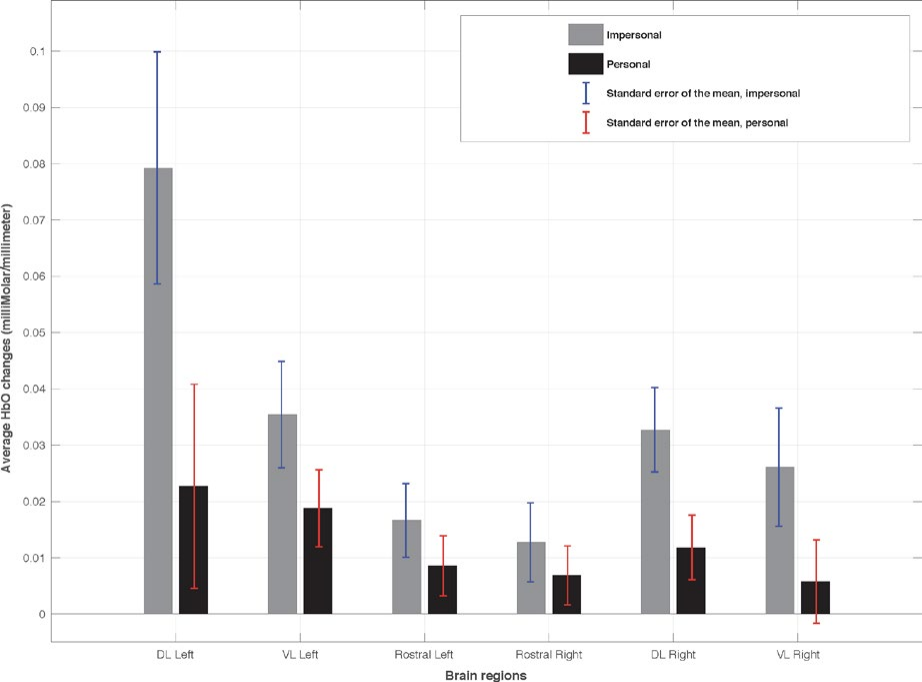
Average HbO changes in approximate prefrontal brain regions for personal and impersonal dilemmas

**Figure 4:**
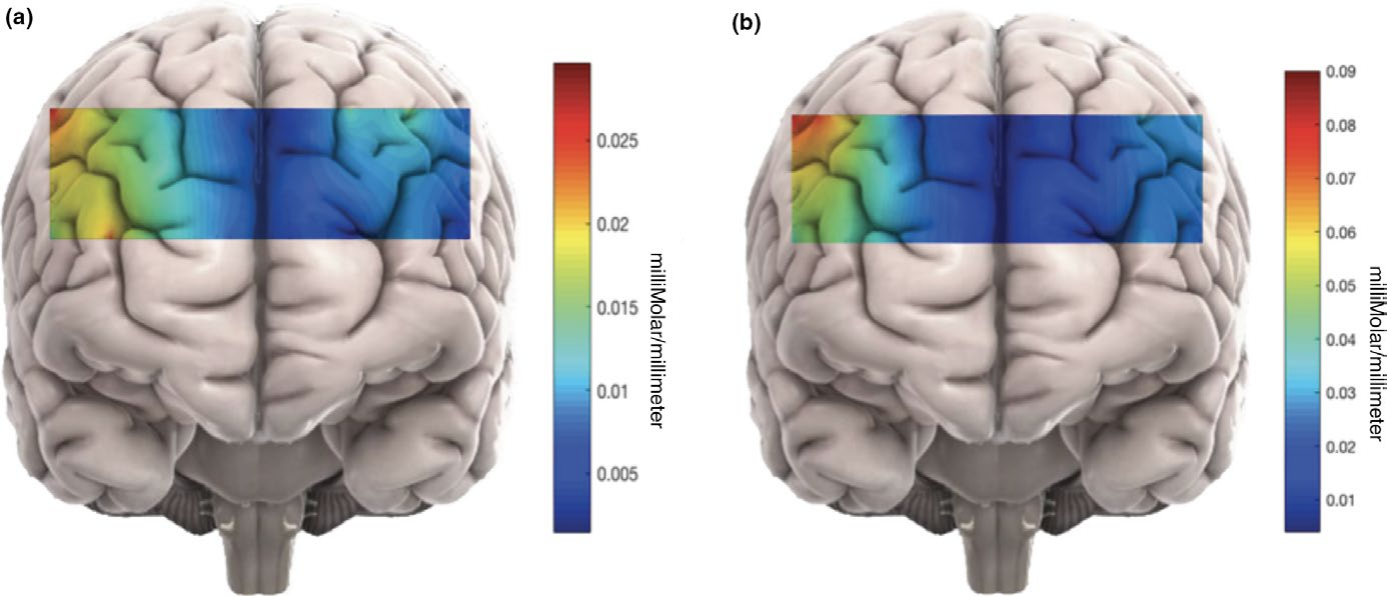
Changes in mean HbO which have been approximately mapped on different brain regions during (a) personal and (b) impersonal MJ. The captured brain activity during impersonal scenarios was significantly higher than personal dilemmas. The average hemodynamic change in the left DL‐PFC for impersonal dilemmas was especially large

The average changes in hemodynamic response in approximate prefrontal areas during personal and impersonal dilemmas are shown in Figure 3 and 4. Note the large difference in average HbO in the left DL‐PFC in Figure 3. Table 1 shows only significant results for the fixed effects analyses, and Tables 2, 3, 4, 5 depict only significant results of post hoc analyses.

**Table 2:** Significant comparisons of average HbO changes in personal versus impersonal categories of moral dilemmas in different PFC regions

Category × Region	*z*‐value	Pr (>|*z*|)
Right DL personal (0.0182) versus left DL impersonal (0.0696)	−3.735	0.0105*
Left VM personal (0.0106) versus left DL impersonal (0.0696)	−4.270	<0.01**
Right VM personal (0.0109) versus left DL impersonal (0.0696)	−4.260	<0.01**
Left VL personal (0.0216) versus Left DL impersonal (0.0696)	−3.483	0.0240*
Right VL personal (0.0093) versus left DL impersonal (0.0696)	−4.384	<0.01***

Notes

Post hoc analysis: Simultaneous tests for general linear hypothesis. Adjusted *p* values, single‐step method. Only results with *p* < 0.05 are reported.

*p < 0.05.

**p < 0.01.

***p < 0.001.

**Table 3:** Significant comparisons of average HbO changes in PFC regions considering responses for personal versus impersonal MJ

Row	Category × Region × Response	*z*‐value	Pr (>|*z*|)
A	Utilitarian left DL personal (−0.0028) versus nonutilitarian Left DL impersonal (0.0784)	−3.830	0.0262*
B	Utilitarian right VM personal (−0.0007) versus nonutilitarian left DL impersonal (0.0784)	−3.730	0.0355*
C	Nonutilitarian right VL personal (0.0109) versus nonutilitarian left DL impersonal (0.0784)	−3.737	0.0354*

Note

Post hoc analysis: Simultaneous tests for general linear hypothesis. Adjusted *p* values, single‐step method.

*p < 0.05.

**p < 0.01.

***p < 0.001.

**Table 4:** Significant comparisons of average HbO changes in different PFC regions during impersonal dilemmas

Region	*z*‐value	Pr (>|*z*|)
Right DL (0.0257) versus Left DL (0.0696)	−2.964	0.03601*
Left VM (0.0149) versus Left DL (0.0696)	−3.695	0.00302**
Right VM (0.0116) versus Left DL (0.0696)	−3.915	0.00120**
Right VL (0.0183) versus Left DL (0.0696)	−3.463	0.00698**

Notes

Post hoc analysis: simultaneous tests for general linear hypothesis. Adjusted *p* values, single‐step method. Only results with *p* < 0.05 are reported.

*p < 0.05.

**p < 0.01.

***p < 0.001.

**Table 5:** Significant comparisons of average HbO changes in different PFC regions considering responses during impersonal dilemmas

Region × Response	z‐value	Pr (>|*z*|)
Left VM, utilitarian (0.0102) versus left DL, nonutilitarian (0.0784)	−3.889	<0.01**
Right VM, utilitarian (0.0117) versus left DL, nonutilitarian (0.0784)	−3.821	<0.01**
Right VL, utilitarian (0.0296) versus left DL, nonutilitarian (0.0784)	−3.349	0.0386*

Notes

Post hoc analysis: simultaneous tests for general linear hypothesis. Adjusted *p* values, single‐step method. Only results with *p* < 0.05 are reported.

*p < 0.05.

**p < 0.01.

***p < 0.001.

HbO changes in the PFC were significantly lower for personal versus impersonal MJ (Table 1, row A). In the model with category and region interaction as fixed effects (Table 1, row B), we saw significant differences between HbO changes in the different PFC regions during personal versus impersonal MJ.

Post hoc analyses indicated that left DL‐PFC activation during impersonal dilemmas was significantly greater than that of all other PFC regions in personal dilemmas (Table 2). Adding utilitarian and nonutilitarian response effects to the model, higher activation occurred during nonutilitarian responses (Table 1, row C, and Table 3).

When the category was personal and the model considered only HbO changes during personal scenarios and region as fixed effects, the average changes of HbO in different prefrontal areas were not significantly different. Consequently, we did not include this case in Table 1. HbO changes for utilitarian responses were significantly higher than nonutilitarian responses during personal scenarios (Table 1, row D). In the model with only region as a fixed effect for HbO changes of impersonal scenarios, the left DL‐PFC had significant HbO changes compared to that of the other prefrontal regions (Table 1, row E, and Table 4).

Utilitarian and nonutilitarian responses to the impersonal scenarios (fixed effect only response) did not have a significant effect on the average HbO changes. When region was added as a fixed effect, the changes in HbO during impersonal scenarios were significantly different in the different PFC regions (Table 1, row F). An interesting observation was the large increase in average HbO in the left DL‐PFC that occurred for nonutilitarian responses (Table 5).

## 4 DISCUSSION

In this study, we monitored the prefrontal activity of 33 healthy adults through fNIRS while they were engaged in personal/impersonal moral dilemmas. Our goal was to examine fNIRS sensitivity to the MJ task and link the different regions of the PFC to the types of scenarios and responses of this task.

First and foremost, we found greater average HbO changes in the whole PFC, and a significantly large change in the left DL‐PFC, for impersonal MJ dilemmas compared to personal ones (Figures 3 and 4, Table 1, rows A and B, and Table 2). This is consistent with previous fMRI studies indicating the brain exhibits differential patterns of activation during these different scenarios (Blair, 1995; Greene et al., 2001). Specifically, one study (Greene et al., 2001) found that brain areas associated with cognitive processes and working memory exhibited greater activity during moral impersonal scenarios than personal scenarios. This was confirmed in a study conducted by Han et al. (2014). Previously, (Glenn et al., 2010, 2009 ; Greene et al., 2004; Han et al., 2014; Hutcherson et al., 2015; Jeurissen, Sack, Roebroeck, Russ, & Pascual‐Leone, 2014) also found greater activation in the DL‐PFC during moral decision‐making and (Greene, 2007; Greene et al., 2001) emphasized the role of the VM‐PFC in emotional decision‐making.

We also found that the HbO differences were significantly different in only three regions when comparing between utilitarian and nonutilitarian responses (Table 1, row C). Nonutilitarian responses to impersonal dilemmas led to the highest activation in the left DL‐PFC, whereas utilitarian responses to personal dilemmas led to the least activation in the left DL‐PFC and right VM‐PFC (Table 3, rows A and B). This is consistent with previous literature indicating more logical thinking (utilitarian) activates the right DL‐PFC the most during personal scenarios, and other regions exhibit less activation (Dashtestani et al., 2018; Greene et al., 2004, 2001 ; Jeurissen et al., 2014). Additionally, nonutilitarian responses led to the least activation in the right VL‐PFC during personal cases (Table 3, row C). This is in agreement with previous studies emphasizing nonutilitarian (more emotional) thinking would invoke the VM‐PFC the most and lateral PFC the least (Greene, 2007; Greene et al., 2004, 2001 ).

Considering only impersonal MJ scenarios, there was relatively less activation in the VM‐PFC compared to the DL‐PFC (Table 4). This is also consistent with previous findings since the medial PFC is responsible for processing emotionally salient events (Greene et al., 2004; Han et al., 2016; Koenigs et al., 2007; Shenhav & Greene, 2014) and it is expected to exhibit lower neural activity during less emotional impersonal dilemmas. The highest activation in the left DL‐PFC occurred for nonutilitarian responses (Table 5). This may indicate that participants were thinking about the outcome logically, thereby involving the DL‐PFC, rather than emotionally. Although DL‐PFC has been mentioned and established as a region more responsible for logical than emotional decision‐making, to our knowledge, no study before ours has reported that the left DL‐PFC is recruited the most during nonutilitarian impersonal decision‐making.

As mentioned earlier, MJ neuroimaging studies have sometimes replicated and support each other’s results or reported new observations that complement them. Therefore, each piece of information is one step toward filling out a part of the neural correlates of human MJ decision‐making puzzle. For instance, Han H. et al., in a study of cultural effects on moral decision‐making (utilizing fMRI) concluded that Korean participants compared to Americans had increased activity in the right DL‐PFC during utilitarian personal MJ. Those authors considered this due to the Koreans stronger need to take cognitive control over their emotional intuitive feelings (Han et al., 2014). In other words, logical thinking during emotional MJ decision‐making (personal) elicits higher functional activity over the right DL‐PFC area. Considering these results along with ours, we may claim that the right DL‐PFC is the most responsible part in utilitarian personal decision‐making (logical thinking in emotional MJ), whereas the left DL‐PFC is highly activated during nonutilitarian impersonal MJ (emotional thinking in logical MJ). Noticeably, DL‐PFC is involved with more rational reasoning, but emotions also play a role in the functional activity in the DL‐PFC. This contradicts the hypothesis that emotional and logical processes are competing systems during MJ decision‐making. Obviously, these interesting findings need to be extensively investigated in the future.

There are some limitations to this study. Although fNIRS is cost effective and user friendly, its limited depth penetration prevents it from assessing critical information beyond the cortex (Homae et al., 2010; Koizumi et al., 2003; Sano, Tsuzuki, Dan, & Watanabe, 2012). Therefore, fMRI remains the gold standard in functional neuroimaging due to its superior spatial resolution and high signal to noise ratio, while fNIRS provides an option to assess hemodynamic information on oxyhemoglobin (HbO) and deoxyhemoglobin (HbR) levels during tasks in which fMRI is not feasible (Yuan, 2013). In addition, our sample size was fairly small (33 subjects) and the number of trials per subject (21 personal and 14 impersonal, total of 35) resulted in only moderate statistical power. Although performing power analysis prior to subject recruitment provides information on what should be expected as scientifically meaningful difference, in this study, we focused on fNIRS feasibility to explore the brain activation in context of MJ decision‐making. Since this has not been widely investigated, lack of previous studies can be another reason of not havening an early estimation on effective sample size (Suresh & Chandrashekara, 2012). Thus, in this paper, we tried to interpret our results with extra cautiousness and we emphasize that further investigations need to be conducted validating our results. Finally, the inability of fNIRS to exactly map the location of brain activation is another limitation. The coregistration in fMRI is done using the anatomical images acquired by structural MRI. Unfortunately, an anatomical dataset or an established standard anatomical system does not exist for fNIRS dataset and needs to be developed.

## 5 CONCLUSION

fNIRS is a noninvasive, affordable, patient‐friendly, and easily applied neuroimaging modality that assesses hemodynamic information about HbO and HbR during cognitive tasks. In spite of its limitations, what it lacks in data acquisition capacity compared to fMRI can make up for in convenience, as it is suited for monitoring brain activity in a wider variety of tasks, patient populations, and settings (Kopton & Kenning, 2014; Strangman et al., 2002). In addition, similar to EEG, cortical hemodynamic information can still be used to characterize cognitive processes (Homae et al., 2010; Koizumi et al., 2003; Sano et al., 2012). In the present study, we evaluated fNIRS as an alternative to fMRI for measuring functional activity recruited during judgment of moral dilemmas. Our results demonstrate the ability of fNIRS to capture patterns of hemodynamic activity associated with various aspects of MJ decision‐making based on the characteristics of the dilemmas presented. Therefore, it can be used to monitor neural activity during dilemmas that differ based on their emotionally saliency, especially when quantitative assessment of brain neural activity in an unusual environment or group of subjects such as children is critical.

Our study goes beyond commonly used self‐report questionnaires. We demonstrated activity in the PFC during MJ decision‐making. Additionally, we found that specific brain regions are active during personal and impersonal MJ scenarios, while considering the type of the responses (utilitarian vs. nonutilitarian) to these dilemmas. Specifically, we found that brain functional activity is significantly higher during nonutilitarian impersonal MJ. Although previous studies have associated DL‐PFC with cognitive processes (Glenn et al., 2010, 2009; Greene et al., 2004; Hutcherson et al., 2015), none has reported that emotional response to more logical (and less emotional) MJ would involve this region as well. Therefore, this may support the belief that rational and emotional processes are intertwined, but contradicts the idea that DL‐PFC is responsible only for logical thinking. However, considering the heterogeneous nature of human MJ in everyday life and the related neural mechanisms, further studies need to be done to validate the results.

## CONFLICT OF INTEREST

None declared.

## References

[brb31116-bib-0001] Anderson, A. A. , Smith, E. , Chowdhry, F. A. , Thurm, A. , Condy, E. , Swineford, L. , … Gandjbakhche, A. H. (2017). Prefrontal hemodynamics in toddlers at rest: A pilot study of developmental variability. Frontiers in Neuroscience, 11, 300 10.3389/fnins.2017.00300. 28611578 PMC5447733

[brb31116-bib-0002] Bates, D. , Mächler, M. , Bolker, B. , & Walker, S. (2014). Fitting linear mixed‐effects models using lme4. arXiv preprint arXiv:1406.5823.

[brb31116-bib-0003] Bates, D. , Maechler , M. , & Bolker , B. (2007). The lme4 package. R Package Version, 2(1), 74.

[brb31116-bib-0004] Bauernfeind, G. , Wriessnegger, S. C. , Daly, I. , & Müller‐Putz, G. R. (2014). Separating heart and brain: On the reduction of physiological noise from multichannel functional near‐infrared spectroscopy (fNIRS) signals. Journal of Neural Engineering, 11(5), 056010 10.1088/1741-2560/11/5/056010 25111822

[brb31116-bib-0005] Blair, R. J. R. (1995). A cognitive developmental approach to morality: Investigating the psychopath. Cognition, 57(1), 1–29. 10.1016/0010-0277(95)00676-P 7587017

[brb31116-bib-0006] Borg, J. S. , Hynes, C. , Van Horn, J. , Grafton, S. , & Sinnott‐Armstrong, W. (2006). Consequences, action, and intention as factors in moral judgments: An fMRI investigation. Journal of Cognitive Neuroscience, 18(5), 803–817. 16768379 10.1162/jocn.2006.18.5.803

[brb31116-bib-0007] Bretz, F. , Hothorn, T. , & Westfall, P. (2016). Multiple comparisons using R. Boca Raton, FL: CRC Press.

[brb31116-bib-0008] Contreras‐Rodríguez, O. , Pujol, J. , Batalla, I. , Harrison, B. J. , Soriano‐Mas, C. , Deus, J. , … Cardoner, N. (2015). Functional connectivity bias in the prefrontal cortex of psychopaths. Biological Psychiatry, 78(9), 647–655. 10.1016/j.biopsych.2014.03.007 24742618

[brb31116-bib-0009] Dashtestani, H. , Zaragoza, R. , Kermanian, R. , Knutson, K. M. , Halem, M. , Anderson, A. , & Gandjbakhche, A. (2018). Importance of left dorsolateral prefrontal cortex in moral judgment using functional near‐infrared spectroscopy In Microscopy Histopathology and Analytics (pp. JW3A‐52). Optical Society of America.

[brb31116-bib-0010] Eres, R. , Louis, W. R. , & Molenberghs, P. (2018). Common and distinct neural networks involved in fMRI studies investigating morality: An ALE meta‐analysis. Social Neuroscience, 13(4), 384–398. 10.1080/17470919.2017.1357657 28724332

[brb31116-bib-0011] Fede, S. J. , Borg, J. S. , Nyalakanti, P. K. , Harenski, C. L. , Cope, L. M. , Sinnott‐Armstrong, W. , … Kiehl, K. A. (2016). Distinct neuronal patterns of positive and negative moral processing in psychopathy. Cognitive, Affective, & Behavioral Neuroscience, 16(6), 1074–1085. 10.3758/s13415-016-0454-z PMC6792390 27549758

[brb31116-bib-0012] Foot, P. (1978). Hume on moral judgment. Her virtues and vices. Oxford, UK: Blackwell.

[brb31116-bib-0013] Franceschini, M. A. , Fantini, S. , Thompson, J. H. , Culver, J. P. , & Boas, D. A. (2003). Hemodynamic evoked response of the sensorimotor cortex measured noninvasively with near‐infrared optical imaging. Psychophysiology, 40(4), 548–560. 10.1111/1469-8986.00057 14570163 PMC3786740

[brb31116-bib-0014] Gao, Y. , & Tang, S. (2013). Psychopathic personality and utilitarian moral judgment in college students. Journal of Criminal Justice, 41(5), 342–349. 10.1016/j.jcrimjus.2013.06.012

[brb31116-bib-0015] Geurts, D. E. , von Borries, K. , Volman, I. , Bulten, B. H. , Cools, R. , & Verkes, R. J. (2016). Neural connectivity during reward expectation dissociates psychopathic criminals from non‐criminal individuals with high impulsive/antisocial psychopathic traits. Social Cognitive and Affective Neuroscience, 11(8), 1326–1334. 10.1093/scan/nsw040 27217111 PMC4967802

[brb31116-bib-0016] Glenn, A. L. , Koleva, S. , Iyer, R. , Graham, J. , & Ditto, P. H. (2010). Moral identity in psychopathy. Judgment and Decision Making, 5(7), 497.

[brb31116-bib-0017] Glenn, A. L. , Raine, A. , & Schug, R. A. (2009). The neural correlates of moral decision‐making in psychopathy. Molecular Psychiatry, 14(1), 5–6. 10.1038/mp.2008.104 19096450

[brb31116-bib-0018] Glenn, A. L. , Raine, A. , Schug, R. A. , Young, L. , & Hauser, M. (2009). Increased DLPFC activity during moral decision‐making in psychopathy. Molecular Psychiatry, 14(10), 909 10.1038/mp.2009.76 19096450

[brb31116-bib-0019] Greene, J. D. (2007). Why are VMPFC patients more utilitarian? A dual‐process theory of moral judgment explains. Trends in Cognitive Sciences, 11(8), 322–323; author reply 323–4. 10.1016/j.tics.2007.06.004. 17625951

[brb31116-bib-0020] Greene, J. D. , Nystrom, L. E. , Engell, A. D. , Darley, J. M. , & Cohen, J. D. (2004). The neural bases of cognitive conflict and control in moral judgment. Neuron, 44(2), 389–400. 10.1016/j.neuron.2004.09.027 15473975

[brb31116-bib-0021] Greene, J. D. , Sommerville, R. B. , Nystrom, L. E. , Darley, J. M. , & Cohen, J. D. (2001). An fMRI investigation of emotional engagement in moral judgment. Science, 293(5537), 2105–2108. 10.1126/science.1062872 11557895

[brb31116-bib-0022] Greve, D. , Goldenholz, D. , Kaskhedikar, G. , Polimeni, J. , Moran, L. , & Schwartz, C. (2009). BOLD physiological noise reduction using spatio‐spectral‐temporal correlations with NIRS. Proceedings of the International Society for Magnetic Resonance in Medicine, 17, 1593.

[brb31116-bib-0023] Han, H. (2017). Neural correlates of moral sensitivity and moral judgment associated with brain circuitries of selfhood: A meta‐analysis. Journal of Moral Education, 46(2), 97–113. 10.1080/03057240.2016.1262834

[brb31116-bib-0024] Han, H. , Chen, J. , Jeong, C. , & Glover, G. H. (2016). Influence of the cortical midline structures on moral emotion and motivation in moral decision‐making. Behavioural Brain Research, 302, 237–251. 10.1016/j.bbr.2016.01.001 26772629

[brb31116-bib-0025] Han, H. , Chen, J. , Jeong, C. , & Glover, G. H. (2015). Relationship between brain regions associated with self and moral functioning in moral judgment. Organ. Hum. Brain Mapp.

[brb31116-bib-0026] Han, H. , Glover, G. H. , & Jeong, C. (2014). Cultural influences on the neural correlate of moral decision making processes. Behavioural Brain Research, 259, 215–228. 10.1016/j.bbr.2013.11.012 24263193

[brb31116-bib-0027] Heekeren, H. R. , Wartenburger, I. , Schmidt, H. , Schwintowski, H. P. , & Villringer, A. (2003). An fMRI study of simple ethical decision‐making. Neuroreport, 14(9), 1215–1219. 10.1097/00001756-200307010-00005 12824762

[brb31116-bib-0028] Hiraoka, M. , Firbank, M. , Essenpreis, M. , Cope, M. , Arridge, S. R. , van der Zee, P. , & Delpy, D. T. (1993). A Monte Carlo investigation of optical pathlength in inhomogeneous tissue and its application to near‐infrared spectroscopy. Physics in Medicine and Biology, 38(12), 1859 10.1088/0031-9155/38/12/011 8108489

[brb31116-bib-0029] Homae, F. , Watanabe, H. , Otobe, T. , Nakano, T. , Go, T. , Konishi, Y. , & Taga, G. (2010). Development of global cortical networks in early infancy. Journal of Neuroscience, 30(14), 4877–4882. 10.1523/JNEUROSCI.5618-09.2010 20371807 PMC6632781

[brb31116-bib-0030] Hoshi, Y. (2003). Functional near‐infrared optical imaging: Utility and limitations in human brain mapping. Psychophysiology, 40(4), 511–520. 10.1111/1469-8986.00053 14570159

[brb31116-bib-0031] Hutcherson, C. A. , Montaser‐Kouhsari, L. , Woodward, J. , & Rangel, A. (2015). Emotional and utilitarian appraisals of moral dilemmas are encoded in separate areas and integrated in ventromedial prefrontal cortex. Journal of Neuroscience, 35(36), 12593–12605. 10.1523/JNEUROSCI.3402-14.2015 26354924 PMC4563040

[brb31116-bib-0032] Jeurissen, D. , Sack, A. T. , Roebroeck, A. , Russ, B. E. , & Pascual‐Leone, A. (2014). TMS affects moral judgment, showing the role of DLPFC and TPJ in cognitive and emotional processing. Frontiers in Neuroscience, 8, 18 10.3389/fnins.2014.00018. 24592204 PMC3923146

[brb31116-bib-0033] Jonathan , H. (2003). The moral emotions In Davidson R. J., Scherer K., & Goldsmith H. H. (Eds.), Handbook of affective sciences (pp. 852–870). Oxford, UK: Oxford University Press.

[brb31116-bib-0034] Karamzadeh, N. , Amyot, F. , Kenney, K. , Anderson, A. , Chowdhry, F. , Dashtestani, H. , … Gandjbakhche, A. H. (2016). A machine learning approach to identify functional biomarkers for traumatic brain injury (TBI) using functional near‐infrared spectroscopy (fNIRS). Brain and behavior, 6(11), e00541 10.1002/brb3.541 27843695 PMC5102640

[brb31116-bib-0035] Koenigs, M. , Kruepke, M. , Zeier, J. , & Newman, J. P. (2011). Utilitarian moral judgment in psychopathy. Social Cognitive and Affective Neuroscience, 7(6), 708–714. 10.1093/scan/nsr048 21768207 PMC3427868

[brb31116-bib-0036] Koenigs, M. , Young, L. , Adolphs, R. , Tranel, D. , Cushman, F. , Hauser, M. , & Damasio, A. (2007). Damage to the prefrontal cortex increases utilitarian moral judgements. Nature, 446(7138), 908 10.1038/nature05631 17377536 PMC2244801

[brb31116-bib-0037] Koizumi, H. , Yamamoto, T. , Maki, A. , Yamashita, Y. , Sato, H. , Kawaguchi, H. , & Ichikawa, N. (2003). Optical topography: Practical problems and new applications. Applied Optics, 42(16), 3054–3062. 10.1364/AO.42.003054 12790457

[brb31116-bib-0038] Kopton, I. M. , & Kenning, P. (2014). Near‐infrared spectroscopy (NIRS) as a new tool for neuroeconomic research. Frontiers in Human Neuroscience, 8, 549 10.3389/fnhum.2014.00549 25147517 PMC4124877

[brb31116-bib-0039] Krueger, C. , & Tian, L. (2004). A comparison of the general linear mixed model and repeated measures ANOVA using a dataset with multiple missing data points. Biological Research for Nursing, 6(2), 151–157. 10.1177/1099800404267682 15388912

[brb31116-bib-0040] Lindenberger, U. , Li, S. C. , Gruber, W. , & Müller, V. (2009). Brains swinging in concert: Cortical phase synchronization while playing guitar. BMC Neuroscience, 10(1), 22 10.1186/1471-2202-10-22 19292892 PMC2662862

[brb31116-bib-0041] McKendrick, R. , Ayaz, H. , Olmstead, R. , & Parasuraman, R. (2014). Enhancing dual‐task performance with verbal and spatial working memory training: Continuous monitoring of cerebral hemodynamics with NIRS. Neuroimage, 85, 1014–1026. 10.1016/j.neuroimage.2013.05.103 23727530

[brb31116-bib-0042] Minati, L. , Visani, E. , Dowell, N. G. , Medford, N. , & Critchley, H. D. (2011). Variability comparison of simultaneous brain near‐infrared spectroscopy and functional magnetic resonance imaging during visual stimulation. Journal of Medical Engineering & Technology, 35(6–7), 370–376. 10.3109/03091902.2011.595533 21780948 PMC3182558

[brb31116-bib-0043] Moll, J. , & de Oliveira‐Souza, R. (2007). Moral judgments, emotions and the utilitarian brain. Trends in Cognitive Sciences, 11(8), 319–321. 10.1016/j.tics.2007.06.001 17602852

[brb31116-bib-0044] Oldfield, R. C. (1971). The assessment and analysis of handedness: The Edinburgh inventory. Neuropsychologia, 9(1), 97–113. 10.1016/0028-3932(71)90067-4 5146491

[brb31116-bib-0045] Pfeifer, M. , Scholkmann, F. , & Labruyère, R. (2017). Signal processing in functional near‐infrared spectroscopy (fNIRS): Methodological differences lead to different statistical results. Frontiers in Human Neuroscience, 11, 641. 29358912 10.3389/fnhum.2017.00641 PMC5766679

[brb31116-bib-0046] Prehn, K. , Wartenburger, I. , Mériau, K. , Scheibe, C. , Goodenough, O. R. , Villringer, A. , … Heekeren, H. R. (2007). Individual differences in moral judgment competence influence neural correlates of socio‐normative judgments. Social Cognitive and Affective Neuroscience, 3(1), 33–46. 10.1093/scan/nsm037 19015093 PMC2569820

[brb31116-bib-0047] Sano, T. , Tsuzuki, D. , Dan, I. , & Watanabe, E. (2012). Adaptive hemodynamic response function to optimize differential temporal information of hemoglobin signals in functional near‐infrared spectroscopy. Complex medical engineering (CME), 2012 ICME international conference on. 2012. IEEE.

[brb31116-bib-0048] Sato, H. , Yahata, N. , Funane, T. , Takizawa, R. , Katura, T. , Atsumori, H. , … Kasai, K. (2013). A NIRS–fMRI investigation of prefrontal cortex activity during a working memory task. Neuroimage, 83, 158–173. 10.1016/j.neuroimage.2013.06.043 23792984

[brb31116-bib-0049] Schaalje, G. B. , McBride, J. B. , & Fellingham, G. W. (2002). Adequacy of approximations to distributions of test statistics in complex mixed linear models. Journal of Agricultural, Biological, and Environmental Statistics, 7(4), 512–524. 10.1198/108571102726

[brb31116-bib-0050] Seara‐Cardoso, A. , Dolberg, H. , Neumann, C. , Roiser, J. P. , & Viding, E. (2013). Empathy, morality and psychopathic traits in women. Personality and Individual Differences, 55(3), 328–333. 10.1016/j.paid.2013.03.011

[brb31116-bib-0051] Shenhav, A. , & Greene, J. D. (2014). Integrative moral judgment: Dissociating the roles of the amygdala and ventromedial prefrontal cortex. Journal of Neuroscience, 34(13), 4741–4749. 10.1523/JNEUROSCI.3390-13.2014 24672018 PMC6608126

[brb31116-bib-0052] Sherafati, A. , Eggebrecht, A. T. , Bergonzi, K. M. , Burns‐Yocum, T. M. , & Culver, J. P. (2018). Improvements in functional diffuse optical tomography maps by global motion censoring techniques Optics and the brain (pp. JW3A–51). Optical Society of America.

[brb31116-bib-0053] Stiratelli, R. , Laird, N. , & Ware, J. H. (1984). Random‐effects models for serial observations with binary response. Biometrics, 961–971. 10.2307/2531147 6534418

[brb31116-bib-0054] Strait, M. , & Scheutz, M. (2014). Using functional near infrared spectroscopy to measure moral decision‐making: Effects of agency, emotional value, and monetary incentive. Brain‐Computer Interfaces, 1(2), 137–146. 10.1080/2326263X.2014.912886

[brb31116-bib-0055] Strangman, G. , Culver, J. P. , Thompson, J. H. , & Boas, D. A. (2002). A quantitative comparison of simultaneous BOLD fMRI and NIRS recordings during functional brain activation. Neuroimage, 17(2), 719–731. 10.1006/nimg.2002.1227 12377147

[brb31116-bib-0056] Suresh, K. , & Chandrashekara, S. (2012). Sample size estimation and power analysis for clinical research studies. Journal of Human Reproductive Sciences, 5(1), 7 10.4103/0974-1208.97779 22870008 PMC3409926

[brb31116-bib-0057] Thomson, J. J. (1986). Rights, resolution, and risk: Essays in moral theory. Cambridge, MA: Harvard University Press.

[brb31116-bib-0058] Tukey, J. W. (1949). Comparing individual means in the analysis of variance. Biometrics, 99–114. 10.2307/3001913 18151955

[brb31116-bib-0059] Xu, R. (2003). Measuring explained variation in linear mixed effects models. Statistics in Medicine, 22(22), 3527–3541. 10.1002/sim.1572 14601017

[brb31116-bib-0060] Yang, Y. , Narr, K. L. , Baker, L. A. , Joshi, S. H. , Jahanshad, N. , Raine, A. , & Thompson, P. M. (2015). Frontal and striatal alterations associated with psychopathic traits in adolescents. Psychiatry Research: Neuroimaging, 231(3), 333–340. 10.1016/j.pscychresns.2015.01.017 PMC4871259 25676553

[brb31116-bib-0061] Yoder, K. , Harenski, C. , Kiehl, K. A. , & Decety, J. (2015). Neural networks underlying implicit and explicit moral evaluations in psychopathy. Translational Psychiatry, 5(8), e625 10.1038/tp.2015.117 26305476 PMC4564570

[brb31116-bib-0062] Young, L. , Koenigs, M. , Kruepke, M. , & Newman, J. P. (2012). Psychopathy increases perceived moral permissibility of accidents. Journal of Abnormal Psychology, 121(3), 659 10.1037/a0027489 22390288 PMC4603562

[brb31116-bib-0063] Yuan, Z. (2013). Spatiotemporal and time‐frequency analysis of functional near infrared spectroscopy brain signals using independent component analysis. Journal of Biomedical Optics, 18(10), 106011–106011. 10.1117/1.JBO.18.10.106011 24150092

[brb31116-bib-0064] Zhang, M. , Liu, T. , Pelowski, M. , Jia, H. , & Yu, D. (2017). Social risky decision‐making reveals gender differences in the TPJ: A hyperscanning study using functional near‐infrared spectroscopy. Brain and Cognition, 119, 54–63. 10.1016/j.bandc.2017.08.008 28889923

[brb31116-bib-0065] Zhao, K. , Ji, Y. , Li, Y. , & Li, T. (2018). Online removal of baseline shift with a polynomial function for hemodynamic monitoring using near‐infrared spectroscopy. Sensors, 18(1), 312 10.3390/s18010312 PMC5795942 29361729

