# Supplementary material for: The role of prefrontal cortex in a moral judgment task using functional near‐infrared spectroscopy
Source: Brain Behav. 2018 Sep 25;8(11):e01116. doi: 10.1002/brb3.1116 (PMC6236239; doi:10.1002/brb3.1116)
Supplement: Supplementary file 1 [file BRB3-8-e01116-s001.docx]

# Supporting Information Appendix S1

#
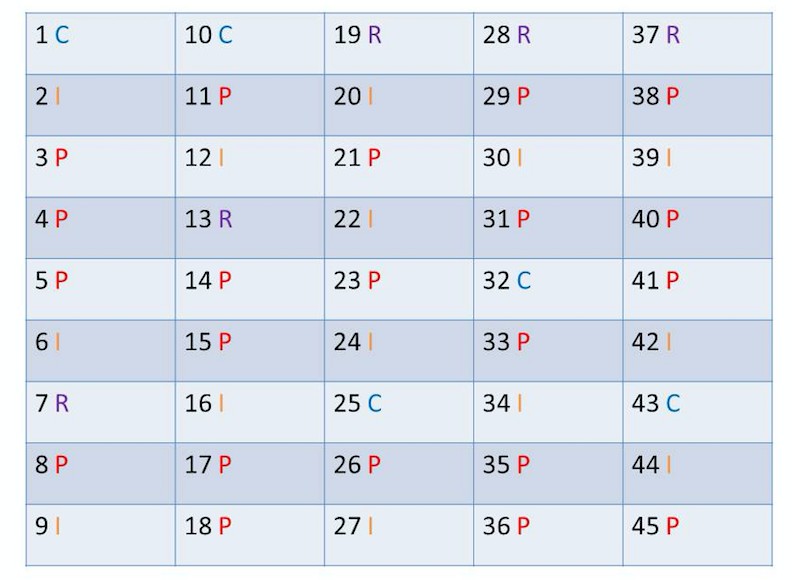
Order of scenarios

Fig. S1. Order of MJ scenarios. “C” = control, “R” = random,“P”= personal, and “I”= impersonal scenarios.

# Example of three slides of a MJ scenario presented to subject

You are at the wheel of a runaway trolley quickly approaching a fork in the tracks. On the tracks extending to the left is a group of five railway workmen. On the tracks extending to the right is a single railway workman.

Press any key to continue

You are at the wheel of a runaway trolley quickly approaching a fork in the tracks. On the tracks extending to the left is a group of five railway workmen. On the tracks extending to the right is a single railway workman.

If you do nothing, the trolley will proceed to the left, causing the deaths of the five workmen. The only way to avoid the deaths of these workmen is to hit a switch on your dashboard that will cause the trolley to proceed to the right, causing the death of the single workman

Press any key to continue

You are at the wheel of a runaway trolley quickly approaching a fork in the tracks. On the tracks extending to the left is a group of five railway workmen. On the tracks extending to the right is a single railway workman.

If you do nothing, the trolley will proceed to the left, causing the deaths of the five workmen. The only way to avoid the deaths of these workmen is to hit a switch on your dashboard that will cause the trolley to proceed to the right, causing the death of the single workman

Would you hit the switch in order to avoid the death of the five workmen?

YES: 1 NO: 2

Fig. S2. Sample of the three slides presented to subjects in our MJ experiment.

# Scenarios

Scenario 1: You’ve decided to buy a raﬄe ticket raﬄing oﬀ Car A and Car B. You only want to buy one raﬄe ticket. You know both cars are equally good. There are more ads for Car B on TV recently and many more people chose to buy tickets for the Car B raﬄe. You have a better chance at winning Car A. Would you buy a ticket for the Car A raffle in order to win a car?

Car A:

-Less People Enter

-Better Chance of Winning

Car B:

-More People Enter

-Less Chance of winning

Scenario 2: You want to make a batch of brownies and find a recipe. The recipe calls for a cup of chopped walnuts. You don’t like walnuts, but you like hazelnuts. You have both walnuts and hazelnuts in your pantry. Is it appropriate to substitute hazelnuts for walnuts in order to avoid eating walnuts?

Brownie 1:

-Walnuts

-You do not like walnuts

Brownie 2

-Hazelnuts

-You like Hazelnuts

Scenario 3: You have a headache. You go to the pharmacy to buy a name-brand headache medicine, but the pharmacy is out of this brand of medication. The pharmacist, whom you trust, tells you that he has in stock a generic product which is, in “exactly the same” as the product you wanted to buy. Is it appropriate to purchase the generic brand instead of the name-brand product?

Name-Brand Medication:

-Intend to buy this originally

Generic Brand Medication:

-Pharmacist says this is “exactly the same”

Scenario 4: A representative of a reputable, national survey organization calls you at home while you are eating dinner. The representative explains that if you are willing to spend half an hour answering questions about a variety of topics, her organization will send you a check for $200. Is it appropriate for you to interrupt your dinner in order to earn $200?

Continue Eating Dinner

Interrupt Dinner

-Answer Survey Questions

-Make $20

Scenario 5: This afternoon, you want to go for a jog and do paperwork. In general, you prefer to get your work done before you exercise.

The weather is nice now, but the weather forecast says that in a few hours it will rain. You dislike jogging in the rain, but you don’t care what the weather is like while you do paperwork. Is it appropriate for you to do your paperwork now and exercise later

Do Paperwork Now:

-Complete paperwork before jogging, which you prefer

-Jog later, but it may rain and you dislike jogging in the rain

Do Paperwork Later:

-Jog before doing paperwork, which you do not prefer

-Jog in nice weather, since later it may rain

Scenario 6: An old friend has invited you to spend the weekend with him. You intend to travel there by car, and there are two routes that you can take: the highway and the coastal road. The highway will get you to your friend’s house faster. The coastal route will get you to your friend’s house slower, but the scenery is breathtakingly beautiful. Is it appropriate for you to take the coastal route in order to observe the beautiful scenery as you drive?

Take the highway:

-Arrive at friend’s house faster

Take the coastal road:

-Arrive at friend’s house slower

-Breathtaking scenery

Moral Judgment Scenarios

Scenario 7: You are at the wheel of a runaway trolley quickly approaching a fork in the tracks. On the lef side of the tracks is a group of ﬁve railway workmen. On the right side of the tracks is a single railway workman. If you do nothing, the trolley will proceed to the left, causing the deaths of the ﬁve workmen. If you hit a switch, the trolley to proceed to the right, causing the death of the single workman. Is it appropriate to hit the switch to avoid the deaths of ﬁve workmen?

Left

side of tracks:

-5 workmen

-If you do not hit the switch, the trolley will kill 5 people

Right side of tracks:

-1 workman

-If you hit the switch, the trolley will kill 1 person

Scenario 8: There is an accident at a hospital and deadly fumes are rising up through the ventilation system. In one room, there are three patients. In another room, there is a single patient. If you do nothing, the fumes will rise up into the room with three patients and cause their deaths. The only way to avoid this is to hit a switch, which will cause the fumes to bypass the room with three patients, but it will enter the room containing the single patient, causing his death. Is it appropriate for you to hit the switch in order to avoid the deaths of the three patients?

Room 1:

-3 patients

-If you do nothing, deadly fumes will rise into this room and kill 3 people

Room 2:

-1 patient

-If you hit a switch, the fumes will bypass room 1, but enter the room with 1 patient, causing his death

Scenario 9: You receive a letter in the mail from a reputable international aid organization asking you to make a donation of $200. This money will provide medical attention to poor people in another part of the world. Is it appropriate for you to not make a donation to this organization in order to save money?

Make a Donation of $200:

-The money will provide medical attention to poor people in another part of the world

Do not make a donation of $200:

-Save Money

Scenario 10: You work for the Bureau of Health, a government agency and you have to decide whether or not to endorse a recently developed vaccine. Most people who take the vaccine develop an immunity, but a small number of people will actually contract the disease. All the available evidence, which is very strong, suggests that the chances of getting the disease due to lack of vaccination are much higher than the chances of getting the disease by taking the vaccine. Is it appropriate for you to direct your agency to encourage the use of this vaccine in order to promote national health?

Endorse Vaccine:

-Most people who receive the vaccine develop immunity

-Evidence suggests the chances of getting the disease without the vaccine are higher than by receiving the vaccine

Do not endorse the vaccine:

-A small number of people who receive the vaccine contract the disease

Scenario 11: You are a member of a government legislature deciding between two different policies concerning environmental hazards. Policy A has a 90% chance of causing no deaths and a 10% chance of causing 1000 deaths. Policy B has a 92% chance of causing no deaths and an 8% chance of causing 10,000 deaths. Is it appropriate for you to vote for Policy A over Policy

B?

Policy A:

-90% Chance of causing no deaths

-10% chance of causing 1000 deaths

Policy B:

-92% chance of causing no deaths

-8% chance of causing 10,000

Scenario 12: You are visiting the sculpture garden of a wealthy art collector overlooking a set of train tracks. A railway workman is working on the tracks, and an empty runaway trolley is heading down the tracks toward the workman. The only way to save the workman’s life is to push one of the art collector’s prized sculptures down into the valley to block the trolley’s passage. Doing this will destroy the sculpture. Is it appropriate for you to destroy the sculpture in order to save this workman’s life?

Push the art collector’s prized sculpture down the valley:

-Save a workman’s life from an empty runaway trolley

-Destroy the sculpture

Do not push the art collector’s prized sculpture down the valley:

-The workman will die from an empty runaway trolley

-The sculpture will not be destroyed

Scenario 13: You are ﬁshing from a seaside dock. Another group boards a small boat and sets sail for a nearby island. Soon after their departure you hear over the radio that there is a violent storm brewing that will affect the other group. The only way that you can ensure their safety is to warn them by borrowing a nearby speedboat. The speedboat belongs to an important ﬁgure who would not take kindly to your borrowing his property. Is it appropriate for you to borrow the speedboat in order to warn the tourists about the storm?

Borrow the speedboat:

-Warn the group on a small boat that a violent storm is brewing

-Get in trouble with an important figure to whom this speed boat belongs

Do not borrow the speedboat:

-The group will not be warned about the storm

-There will be no issues with the important figure

Scenario 14: You are a member of a government legislature deciding between two diﬀerent policies concerning environmental hazards. Policy A has a 90% chance of causing no deaths and a 10% chance of causing 1000 deaths. Policy B has a 92% chance of causing no deaths and an 8% chance of causing 10,000 deaths. Is it appropriate for you to vote for Policy A over Policy B?

Policy A:

-90% Chance of causing no deaths

-10% chance of causing 1000 deaths

Policy B:

-92% chance of causing no deaths

-8% chance of causing 10,000

Scenario 15: You are visiting the sculpture garden of a wealthy art collector overlooking a set of train tracks. A railway workman is working on the tracks, and an empty runaway trolley is heading down the tracks toward the workman. The only way to save the workman’s life is to push one of the art collector’s prized sculptures down into the valley to block the trolley’s passage. Doing this will destroy the sculpture. Is it appropriate for you to destroy the sculpture in order to save this workman’s life?

Push the art collector’s prized sculpture down the valley:

-Save a workman’s life from an empty runaway trolley

-Destroy the sculpture

Do not push the art collector’s prized sculpture down the valley:

-The workman will die from an empty runaway trolley

-The sculpture will not be destroyed

Scenario 16: You are ﬁshing from a seaside dock. Another group boards a small boat and sets sail for a nearby island. Soon after their departure you hear over the radio that there is a violent storm brewing that will aﬀect the other group. The only way that you can ensure their safety is to warn them by borrowing a nearby speedboat. The speedboat belongs to an important ﬁgure who would not take kindly to your borrowing his property. Is it appropriate for you to borrow the speedboat in order to warn the tourists about the storm?

Borrow the speedboat:

-Warn the group on a small boat that a violent storm is brewing

-Get in trouble with an important figure to whom this speed boat belongs

Do not borrow the speedboat:

-The group will not be warned about the storm

-There will be no issues with the important figure

Scenario 17: You are ﬁshing from a seaside dock. Another group boards a small boat and sets sail for a nearby island. Soon after their departure you hear over the radio that there is a violent storm brewing that will aﬀect the other group. The only way that you can ensure their safety is to warn them by borrowing a nearby speedboat. The speedboat belongs to an important ﬁgure who has hired a loyal guard to make sure that no one uses his boat without permission. To get to the speedboat you will have to lie to the guard. Is it appropriate for you to lie to the guard in order to borrow the speedboat and warn the group about the storm?

Lie to the guard to borrow the speedboat owned by an important figure:

-Warn the group on a small boat that a violent storm is coming

Do not lie to the guard to borrow the speedboat owned by an important figure:

-The group will not be warned about the storm

Scenario 18: You are at the wheel of a runaway trolley quickly approaching a fork in the tracks. On the left is a group of ﬁve railway workmen. On the right is a group of seven railway workmen. If you do nothing, the trolley will proceed to the left, causing the deaths of the ﬁve workmen. If you hit a switch, the trolley to proceed to the right, causing the death of seven workman. Is it appropriate to hit the switch to avoid the deaths of ﬁve workmen?

Left side of the tracks:

-5 workmen

-If you do not hit the switch, the trolley will kill 5 people

Right side of the tracks:

-7 workmen

-If you hit the switch, the trolley will kill 7 people

Scenario 19: There is an accident at a hospital and deadly fumes are rising up through the ventilation system. In one room, there are three patients. In another room, there are seven patients. If you do nothing, the fumes will rise up into the room with three patients and cause their deaths. The only way to avoid this is to hit a switch, which will cause the fumes to bypass the room with three patients, but it will enter the room containing seven patients. Is it appropriate for you to hit the switch in order to avoid the deaths of the three patients?

Room 1:

-3 Patients

-If you do nothing, deadly fumes will rise into this room and kill 3 people

Room 2:

-7 patients

-If you hit a switch, the fumes will bypass room 1, but enter the room with 7 patients, causing their deaths.

Scenario 20: You have a friend who is trying to ﬁnd a job without success. He decided to put false information on his resume to make it more impressive. By doing this he managed to get hired, beating out other candidates who were more qualiﬁed than he. Was it appropriate for your friend to put false information on his resume in order to help him ﬁnd employment?

Friend needed a job:

-Put false information on resume

Friend received job:

-Beat other candidates who were more qualified than him

Scenario 21: You are the owner of a small business. You could lower your taxes by pretending that some of your personal expenses are business expenses. Is it appropriate for you to pretend that certain personal expenses are business expenses in order to lower your taxes?

Lie about personal expenses:

-Pretend some personal expenses are business expenses for your small business

Do not lie about personal expenses:

-Lower your taxes

Scenario 22: You are a member of a government legislature deciding between two diﬀerent policies concerning environmental hazards. Policy A has a 90% chance of causing no deaths and a 10% chance of causing 1000 deaths. Policy B has a 92% chance of causing no deaths and an 8% chance of causing 10,000 deaths. Is it appropriate for you to vote for Policy B over Policy A?

Policy A:

-90% chance of causing no deaths

-10% chance of causing 1000 deaths

Policy B:

-92% chance of causing no deaths

-8% chance of causing 10,000 deaths

Scenario 23: You are a member of a government legislature deciding between two diﬀerent policies concerning environmental hazards. Policy A has a 90% chance of causing no deaths and a 10% chance of causing 1000 deaths. Policy B has a 92% chance of causing no deaths and an 8% chance of causing 10,000 deaths. Is it appropriate for you to vote for Policy B over Policy A?

Policy A:

-90% chance of causing no deaths

-10% chance of causing 1000 deaths

Policy B:

-92% chance of causing no deaths

-8% chance of causing 10,000 deaths

Scenario 24: You are a management consultant with access to conﬁdential information that is useful to investors. You have a friend who plays the stock market. You owe this friend a sizable sum of money. By providing her with certain conﬁdential information you could help her make a lot of money. If you did this, she would insist on canceling your debt. Releasing information in this way is strictly forbidden by federal law. Is it appropriate for you to release this information to your friend so that she will cancel your debt?

Give friend confidential information:

-You owe friend money

-This information will allow her to make a lot of money in the stock market

-Releasing this information is illegal

Do not give friend confidential information

-You will still owe your friend money

-You will not break the law

Scenario 25: You are a lawyer working on a big case. The judge presiding over the trial happens to be someone you knew from law school. The two of you were rather friendly back then, but now, decades later, it seems that your old friend barely remembers you. You’re quite sure that if you were to talk to him over lunch, you could jog his memory and he would begin to see you as an old buddy, which would be very good for your work on this case. It’s illegal for judges and lawyers working on the same case to meet socially. Is it appropriate for you to meet with this judge socially in order to help you win your case?

Meet judge for lunch:

-Judge is your old friend from law school

-Meeting with hum for lunch could help your case

-Meeting socially on a case is illegal

Do not meet the judge for lunch:

-You will not break the law

Scenario 26: You are walking down the street when you come across a wallet lying on the ground containing several hundred dollars in cash as well the owner’s driver’s license. It’s clear that the wallet’s owner is wealthy. You, on the other hand, have been hit by hard times and could really use some extra money. You consider sending the wallet back to the owner without the cash, keeping the cash for yourself. Is it appropriate for you to keep the money you found in the wallet in order to have more money for yourself?

Keep the cash:

-The wallet’s owner is wealthy, apparent by the several hundred dollars in his wallet

-You have been hit by hard times and could use the extra money

-Send the wallet back without the cash and only his ID

Do not keep the cash:

-Send it back to the owner

Scenario 27: A runaway trolley is heading down the tracks toward ﬁve workmen who will be killed if the trolley continues. You are on a footbridge over the tracks with a stranger who is very large. The only way to save the lives of the ﬁve workmen is to push this stranger oﬀ the bridge where his large body will stop the trolley. The stranger will die, but the ﬁve workmen will be saved. Would you push the stranger on to the tracks in order to save the ﬁve workmen?

Push a stranger:

-Save the lives of the 5 workmen on the tracks from a runaway trolley

-Kill 1 stranger

Do not push a stranger:

-The 5 workmen on the track will die from a runaway trolley

-You will not kill 1 stranger

Scenario 28: You are a doctor. You have ﬁve patients who will die due to a failing organ. You have another patient who is healthy. The only way that you can save the lives of the ﬁrst ﬁve patients is to transplant ﬁve of this young man’s organs (against his will) into the bodies of the other ﬁve patients. If you do this, the young man will die, but the other ﬁve patients will live.

Give transplant:

-Transplant the organs of one healthy patient into the 5 patients who will die of a failing organ

-The young, healthy patient will die

-You take his organs against his will

Do not give transplant:

-The young, healthy patient will live

-The 5 sick patients will die of failing organs

Scenario 29: You are driving when you hear a plea for help from the roadside. You pull over and encounter a man whose legs are covered with blood. The man had an accident while hiking and asks you to take him to a nearby hospital. Initially you want to help this man, who will probably lose his leg if he does not get to the hospital soon. However, his blood will ruin the leather upholstery of your car. Is it appropriate for you to leave this man by the side of the road in order to preserve your leather upholstery?

Help the man:

-The man had an accident while hiking and cannot walk (his legs are covered in blood)

-The blood will ruin the leather upholstery of your car

Do not help the man:

-The man will probably lose his leg if he does not get to the hospital soon

-The leather upholstery of your car will not be ruined

Scenario 30: You are the leader of a mountaineering expedition that is stranded in the wilderness. Your expedition includes a family of six that has a genetically caused vitamin deﬁciency. A few people’s kidneys contain large amounts of this vitamin. There is one such person in your party. The only way to save the lives of the six members of this family is to remove one of this man’s kidneys so that the necessary vitamins may be extracted from it. The man will not die, but his health will be compromised. The man is opposed to this plan.

Take 1 man’s kidney:

-This kidney contains necessary vitamins that will save the lives of 6 members of a family that have a genetically caused vitamin deficiency

-The man’s health will be compromised

-The man is opposed to this plan

Do not take 1 man’s kidney:

-The 6 family members will die from the vitamin deficiency since you are stranded in the wilderness

-The man with the necessary kidney will not have compromise health

Scenario 31: You are a young architect visiting one of your construction sites with your boss. Your boss makes everyone around him miserable, including you. If you were to push him oﬀ of the building, he would fall to his death and everyone would think it was an accident. Is it appropriate for you to push your boss oﬀ of the building in order to get him out of your life?

Push boss off building:

-Boss makes everyone miserable at work

-Everyone would think it is an accident and that he fell off the building

Do not push boss off building:

-Your boss will live and continue to give everyone a hard time

Scenario 32: You are a young architect visiting one of your construction sites with your boss. Your boss makes everyone around him miserable, including you. If you were to push him oﬀ of the building, he would fall to his death and everyone would think it was an accident. Is it appropriate for you to push your boss oﬀ of the building in order to get him out of your life?

Push boss off building:

-Boss makes everyone miserable at work

-Everyone would think it is an accident and that he fell off the building

Do not push boss off building:

-Your boss will live and continue to give everyone a hard time

Scenario 33: You are the head of a poor household in a developing country. Your crops have failed and there is no way to feed your family. Your sons are too young to work jobs in the city, but your daughter could fare better. You know a man from your village who lives in the city and who makes sexually explicit ﬁlms featuring small children such as your daughter. One year of work could earn enough money to keep your family fed for several growing seasons. Is it appropriate for you to employ your daughter in the child pornography industry in order to feed your family?

Employ your daughter:

-Your crops have failed and you do not have the means to feed your family

-Your sons are too young to work jobs in the city

-Money your daughter makes could feed your family for several growing seasons

Do not employ your daughter:

-She would not have to make sexually explicit films featuring small children

Scenario 34: Enemy soldiers have taken over your village with orders to kill all civilians. You and some of your townspeople are hiding in a cellar. Outside you hear the voices of soldiers. Your baby begins to cry loudly and you cover his mouth. If you remove your hand, the soldiers will hear and kill everyone hiding out in the cellar. To save yourself and the others you must smother your child to death. Is it appropriate for you to smother your child in order to save yourself and the other townspeople?

Remove your hand

-The soldiers will hear and kill everyone hiding in the cellar

Cover your baby’s mouth

-Your child will be smothered to death

Scenario 35: You are a ﬁfteen-year-old girl who has become pregnant. You kept it a secret by gaining weight and wearing loose clothes. One day at school, your water breaks and you deliver the baby in the girl’s locker room. You are not prepared to care for this baby. It would be easiest to clean up, wrap the baby in towels, and throw the baby in a dumpster acting as if nothing had ever happened. Is it appropriate for you to throw your baby in the dumpster in order to move on with your life?

Throw baby in dumpster:

-You are not prepared to take care of the baby

-You kept your pregnancy a secret by gaining weight and weather loose clothes

-If you throw away the baby, you move on with your life

Keep the baby:

-You are unprepared to take care of the baby

-You will save the baby’s life

Scenario 36: You and your family are crossing a mountain range on your return journey to your homeland and you inadvertently set up camp on a local clan’s sacred burial ground. The leader of the clan says that according to the local laws, you and your family must be put to death. However, he will let you live with your husband and other three children if you yourself will kill your oldest son. Is it appropriate for you to kill your oldest son in order to save your husband and your other three children?

Kill your oldest son:

-Save yourself and your three other children

Do not kill your oldest son:

-Your family will be put to death by the local clan Scenario

37: You are the leader of a small group of soldiers on the way back from a mission in enemy territory. One of your men steps in trap and is badly injured. The trap is connected to a radio device alerting the enemy to your presence. If the enemy ﬁnds your injured man they will torture him and kill him. If you try to take him with you your entire group will be captured. The only way to prevent this injured soldier from being tortured is to shoot him yourself. Is it appropriate for you to shoot this soldier in order to prevent him from being tortured by the enemy?

Kill your fellow soldier:

-Prevent him from being tortured by the enemy

Do not kill your fellow soldier:

-If you leave him, he will be tortured by the enemy

-If you take him with you, your whole group will be captured

Scenario 38: You are in hospital lounge waiting to visit a sick friend. A young man sitting next to you explains that his father is very ill and has a week to live. His father has a substantial life insurance policy that expires at midnight. If his father dies before midnight, this young man will receive a very large sum of money. He oﬀers you half a million dollars to go up to his father’s room and smother his father with a pillow. Is it appropriate for you to kill this man’s father in order to get money for yourself and this young man?

Kill man’s father:

-The man’s father is very ill and has a week to live

-He has a substantial life insurance policy that expires at midnight

-The man offers to give you half a million dollars if you smother his father with a pillow

Do not kill man’s father:

-You will not take someone’s life

-You will not receive any money

Scenario 39: Your plane has crashed in the Himalayas. The only survivors are yourself, another man, and a young boy. The three of you travel for days in harsh weather. Your only chance at survival is to ﬁnd your way to small a village on the other side of the mountain, several days away. The boy has a broken leg and cannot move very quickly. His chances of surviving the journey are essentially zero. Without food, you and the other man will probably die as well. The other man suggests that you sacriﬁce the boy and eat his remains over the next few days. Is it appropriate to kill this boy so that you and the other man may survive your journey to safety?

Kill the boy:

-The boy has a broken leg and cannot move very quickly. His chances of surviving the journey are essentially zero.

-If you kill him and eat his remains over the next few days, you may survive unless you can make it to safety Do not kill the boy:

-You will not take his life

-You may not survive your journey to safety

Scenario 40: You have been dissatisﬁed with your marriage for several years because your wife no longer appreciates you. You remember how she appreciated you years ago when you took care of her after she was mugged. You devise a plan to regain your wife’s aﬀection. You will hire a man to break into your house, beat up your wife and rape her. You, upon hearing the horrible news, will take care of her and comfort her, and she will once again appreciate you. Is it appropriate for you to hire a man to rape your wife so that she will appreciate you as you comfort her?

Hire a man to rape your wife

-After hearing the news, you will take care of hear and comfort her and she will appreciate you

Do not hire a man to rape your wife

-Your wife will continue to not appreciate you, like how she appreciated you years ago after she was mugged.

Scenario 41: A young boy is visiting his grandmother. Usually she gives him a gift of a few dollars, but this time she doesn’t. He asks her why not and she says something about how he doesn’t write her as many letters as he is used to. Angered by this, the boy decides to play a trick on her. While his grandmother is busy cooking he removes a handful of pills from the medicine cabinet and puts them in his grandmother’s teapot. Later that night, she makes her tea, and the dissolved pills make her sick. In the morning, she is dead. Was it appropriate for this boy to put pills in his grandmother’s teapot in order to play a trick on her?

Boy put pills in his grandmother’s tea:

-He is angered she did not give him a few dollars

-She gets sick, the next day she is dead

Grandmother does not give the boy money for a gift:

-She says he does not write her as many letters as he used to

Scenario 42: You are on a cruise ship when there is a ﬁre on board, and the ship has to be abandoned. The lifeboats are carrying too many people. You are in a lifeboat that is dangerously low in the water—a few inches lower and it will sink. The seas start to get rough, and the boat ﬁlls with water. If nothing is done the boat will sink. There is an injured person who will not survive in any case. If you throw that person overboard the boat will stay aﬂoat and the remaining passengers will be saved. Is it appropriate for you to throw this person overboard in order to save the lives of the remaining passengers?

Throw person overboard:

-The boat is too heavy and it will sink in the storm

-The man is injured and will not survive

-The remaining passengers will live

Do not throw person overboard:

-The injured man will not be killed

-The remaining passengers may not survive

Scenario 43: You are a waiter. You overhear one of your customers say that he is about to go to jail and he plans to infect as many people as possible with HIV beforehand. You know him well enough to know that he is telling the truth and that he has access to many potential victims. The man has a very strong allergy to poppy seeds. Is it appropriate for you to cause this man to have a serious allergy attack in order to prevent him from spreading HIV?

Cause customer to have allergic reaction to poppy seeds:

-The customer plans to infect as many people with HIV before going to jail

Do not cause customer to have allergic reaction to poppy seeds:

-Many victims may be infected with HIV

Scenario 44: You are part of a group of ecologists who live in a remote stretch of jungle. The entire group, which includes eight children, has been taken hostage by a group of paramilitary terrorists. One of the terrorists takes a liking to you. He informs you that his leader intends to kill you and the rest of the hostages the following morning. He is willing to help you and the children escape, but as an act of good faith he wants you to kill one of your fellow hostages whom he does not like. If you refuse his oﬀer all the hostages including the children and yourself will die. If you accept his oﬀer then the others will die in the morning but you and the eight children will escape. Is it appropriate for you to kill one of your fellow hostages in order to escape from the terrorists and save the lives of the eight children?

Kill a fellow hostage:

-One of the enemy soldiers will help you and 8 children escape

-The other hostages in the camp will die the following morning

Do not kill a fellow hostage:

-You and the children will die

-The other hostages will also die

Scenario 45: You are part of a group of ecologists who live in a remote stretch of jungle. The entire group, which includes eight children, has been taken hostage by a group of paramilitary terrorists. One of the terrorists takes a liking to you. He informs you that his leader intends to kill you and the rest of the hostages the following morning. He is willing to help you and the children escape, but as an act of good faith he wants you to kill one of your fellow hostages whom he does not like. If you refuse his oﬀer all the hostages including the children and yourself will die. If you accept his oﬀer then the others will die in the morning but you and the eight children will escape. Is it appropriate for you to kill one of your fellow hostages in order to escape from the terrorists and save the lives of the eight children?

Kill a fellow hostage:

-One of the enemy soldiers will help you and 8 children escape

-The other hostages in the camp will die the following morning

Do not kill a fellow hostage:

-You and the children will die

-The other hostages will also die

Scenario 46: You are negotiating with a powerful and determined terrorist who is about to set oﬀ a bomb in a crowded area. Your have his teenage son in your custody. In order to stop him, you must contact him over the satellite, break one of his son’s arms over the camera, and then threaten to break the other arm if he does not give himself up. Is it appropriate for you to break the terrorist’s son’s arm in order to prevent the terrorist from killing thousands of people with his bomb?

Break the terrorists’ son’s arm:

-Stop him from setting off a bomb in a crowded area which would kill thousands of people

Do not break his son’s arm:

-You will not hurt an innocent man

-Thousands of people may die from the bomb

Scenario 47: You are the captain of a military submarine travelling underneath a large iceberg. An explosion has caused you to lose most of your oxygen supply and has injured one of your crew who is quickly losing blood. The injured crew member is going to die from his wounds no matter what happens. The remaining oxygen is not suﬃcient for the entire crew to make it to the surface. The only way to save the other crew members is to shoot dead the injured crew member so that there will be just enough oxygen for the rest of the crew to survive. Is it appropriate for you to kill the fatally injured crew member in order to save the lives of the remaining crew members?

Kill the injured crew member:

-Save the oxygen supply for the healthy crew members so they will survive until the submarine can make it to the surface

Do not kill the injured crew member:

-Use up the remaining oxygen and the rest of the crew may not survive

Scenario 48: You are the leader of a small army that consists of warriors from two tribes, the hill tribe and the river tribe. You belong to neither tribe. During the night a hill tribesman got into an argument with a river tribesman and murdered him. The river tribe will attack the hill tribe unless the murderer is put to death, but the hill tribe refuses to kill one of its own warriors. The only way for you to avoid a war is to publicly execute the murderer by cutting off his head with your sword. Is it appropriate for you to cut oﬀ this man’s head in order to prevent the two tribes from ﬁghting a war that will cost hundreds of lives?

Execute the murderer:

-Avoid the breakout of war between two tribes

-You do not belong to either tribe

Do not execute the murderer:

-The river tribe will attack the hill tribe resulting in war

-You do not belong to either tribe

Scenario 49: It is wartime and you and your two children, ages eight and ﬁve, are living in a territory that has been occupied by the enemy. At the enemy’s headquarters is a doctor who performs painful experiments on humans that inevitably lead to death. He intends to perform experiments on one of your children, but he will allow you to choose which of your children will be experimented upon. You have twenty-four hours to bring one of your children to his laboratory. If you refuse to bring one of your children to his laboratory he will ﬁnd them both and experiment on both of them. Is it appropriate for you to bring one of your children to the laboratory in order to avoid having them both die?

Chose one child to bring to the doctor:

-One child will die, the other will live

Do not choose which child for the doctor to do experiments on:

-The doctor will take both children and they will both die

Scenario 50: A viral epidemic has spread across the globe killing millions of people. You have developed two substances in your home laboratory. You know that one of them is a vaccine, but you don’t know which one. You also know that the other one is deadly. Once you ﬁgure out which substance is the vaccine you can use it to save millions of lives. You have with you two people who are under your care, and the only way to identify the vaccine is to inject each of these people with one of the two substances. One person will live, the other will die, and you will be able to start saving lives with your vaccine. Is it appropriate for you to kill one of these people with a deadly injection in order to identify a vaccine that will save millions of lives?

Inject vaccine:

-One patient will live, the other patient will die

-Knowing which substance is the vaccine and which is poison will save millions of people infected

Do not inject vaccine:

-You will not intentionally kill on patient
